# Partitioning patterns of fish and sea urchin herbivory from tropical to temperate reefs

**DOI:** 10.1007/s00442-026-05997-0

**Published:** 2026-10-25

**Authors:** Matthew Rose, Paula A. Ruiz-Ruiz, Sterling B. Tebbett, Tyson R. Jones, Yann Herrera-Fuchs, Lara Denis-Roy, Claire L. Butler, Scott D. Ling

**Affiliations:** Institute for Marine and Antarctic Studies, University of Tasmania, Hobart, Tasmania 7004 Australia

**Keywords:** Coral reefs, Kelp beds, Latitudinal gradients, Ecosystem processes, Shallow reef

## Abstract

Herbivory can structure vegetation and affect the states of sunlit ecosystems. On shallow tropical and temperate reefs, herbivory can inhibit macroalgal dominance and promote turf-dominated benthic states. However, standardised comparisons of tropical and temperate herbivory are primarily focussed on fish, leaving the relative contributions of sea urchins poorly understood. Here, we examined thermal realm (tropical vs temperate) and depth (2, 5, 8 and 12-m) effects on relative herbivory patterns and herbivore distributions, using standardised macroalgal assays (N = 1,340) and underwater visual surveys (N = 67 surveys) at 49 sites across six locations spanning approximately 29° of latitude in eastern Australia. Assays were deployed to partition the relative intensity of herbivory using two partitioning treatments: 1) assays were suspended above the benthos allowing only fish consumption (water-column herbivory), and 2) assays were laid flat on the benthos enabling fish and urchin consumption (benthic herbivory). Assay results showed higher fish herbivory on tropical reefs than on temperate reefs, whereas relative benthic herbivory did not differ between thermal realms. Variation in assay consumption across depth was limited. Results from assays and surveys aligned, with higher herbivorous fish abundance on tropical reefs and higher urchin abundance on temperate reefs. Relative patterns in fish and urchin herbivory between thermal realms underscores differences in how herbivory occurs and where in the water-column it predominates, highlighting a key axis of variation for tropicalizing reefs.

**Supplementary Information:**

The online version contains supplementary material available at https://doi.org/10.1007/s00442-026-05997-0.

## Introduction

Herbivory shapes the structure and function of terrestrial and marine ecosystems (Holling 1992; Bellwood et al. 2019). By consuming vegetation, herbivores transfer energy to higher trophic levels, driving secondary production while structuring vegetation patterns (Horn 1989; Brandl et al. 2019). On land, both large mammals and small insects can mediate successional stages of vegetation from the forest floor to the upper canopy and influence overall forest ecosystem states (Meyer et al. 2011; Ramirez et al. 2018). In the marine environment, nominally herbivorous fish and sea urchins can alter tropical and temperate reef states by inhibiting the dominance of fleshy macroalgae (Carpenter 1986; Steneck et al. 2002; Hughes et al. 2007; Rasher et al. 2012). Indeed, herbivory levels of fish and sea urchins can largely determine the balance between net productivity and consumption on reefs, which can ultimately shape the state of reefs and socio-ecological outcomes (Hughes 1994; Ling et al. 2015).

The balance between production and consumption is not fixed across space as key environmental gradients can regulate both algal productivity and the intensity of herbivory (Steneck 1988; Fox and Bellwood 2007). For example, as water depth increases at the scale of metres across a reef profile, steep gradients in key environmental parameters such as light and swell turbulence occur (Copertino et al. 2005; Laverick et al. 2020; Tebbett et al. 2023), directly and indirectly structuring algal productivity (Copertino et al. 2005; Tebbett and Bellwood 2021) and herbivore distributions (Steneck 1988; Fox and Bellwood 2007; Bejarano et al. 2017). Indeed, some herbivore groups concentrate their foraging efforts in shallow waters, which can increase top-down pressure on shallow algal production (Russ 2003; Tootell and Steele 2016; Kriegisch et al. 2020). On reef zones where intense herbivory exceeds algal production, the functional impact of herbivory becomes most apparent (Carpenter 1990; Russ 2003).

Similarly, across tropical and temperate reefs, the balance between herbivory and algal production can promote specific reef states. On tropical reefs, high herbivory can reduce competitive macroalgae and promote reefs typified by live coral cover and short productive algal turfs (Carpenter 1986; Bellwood et al. 2004). For temperate reefs, when herbivory rates exceed macroalgal production, overconsumption can occur, with sea urchin herbivory providing the most striking example (Steneck et al. 2002; Steneck 2020). The collapse of complex temperate macroalgal habitat as a result of sea urchin overgrazing gives way to the proliferation of persistent urchin barrens and turf cover, with reduced productivity and biodiversity (Filbee-Dexter and Scheibling 2014; Ling et al. 2015; Filbee-Dexter and Wernberg 2018; Eger et al. 2024; Ruiz-Ruiz et al. 2025). Ultimately, fish and urchin herbivory can limit macroalgae on both tropical and temperate reefs, yet standardised comparisons of these two groups remain limited across thermal realms and depth.

Fundamental knowledge gaps in how herbivory occurs on reefs across thermal realms inhibit capacity to predict and respond to the increasing impacts of “tropicalization” (Vergés et al. 2014, 2016; Ripple et al. 2023). “Tropicalization” describes the phenomenon of cascading changes as warm-adapted species redistribute to temperate reefs (Schuster et al. 2022). Redistributions are associated with increased rates of herbivory by novel herbivores, which can result in macroalgal loss across entire reef-scapes (Vergés et al. 2014, 2016; Bennett et al. 2015; Zarco‐Perello et al. 2020). Historically, underwater visual surveys have been used to quantify patterns in the abundance and biomass of herbivorous fish to then infer herbivory levels (Ferreira et al. 2004; Floeter et al. 2004, 2005; Diamond and Roy 2023; Tebbett et al. 2025c), which may fail to represent the process of herbivory itself given that fish can rove over large distances when feeding (Steneck 1983; Tebbett et al. 2024). Nevertheless, some studies have revealed that fish herbivory is higher on tropical reefs compared to temperate reefs (e.g., Poore et al. 2012), including by directly quantifying fish bite rates using remote video monitoring across tropical and temperate systems (e.g., Longo et al. 2014, 2019). While these studies represent fundamental knowledge, they have solely focused on fish and, therefore, only provide a partial insight into how herbivory varies across thermal realms.

To extend standardised measurements of herbivory beyond reef fish, we must account for the contributions of urchins, by utilising methods that consider their behavioural differences. Fish and urchins differ distinctly in their locomotion, food ingestion and diel behaviours, producing distinct differences in their feeding patterns (Ogden and Lobel 1978; Steneck 1988; Flukes et al. 2012; Byrne and Andrew 2020; Jones et al. 2024). While daytime video surveys provide effective estimates of bite rates by herbivorous fish, they fail to represent urchins. To represent urchin herbivory on the benthos, methods must span approximately 24-h to account for elevated urchin activity during nocturnal periods (Flukes et al. 2012; Jones et al. 2024, 2026). Standardised assays partitioning between fish and urchins across biogeographical scales offer a solution (e.g., Díaz-Flores et al. 2026). While not deriving an explicit bite rate, assays do provide rapid approximations of relative herbivory levels between these two herbivore groups (sensu Hay 1981).

To understand the relative contribution of both fish and urchins to herbivory patterns across biogeographical scales, we deployed standardised macroalgal assays that allowed for partial partitioning of the relative contributions of fish and urchins to herbivory on tropical and temperate reefs in Australia and conducted underwater visual surveys to define local herbivore communities. Partitioning the relative contributions of these two herbivore groups was achieved by deploying assays suspended in the water-column that only fish could consume (fish herbivory) alongside assays that both fish and urchins could consume (benthic herbivory). Additionally, because depth has a strong influence on primary production, algal structure and in turn herbivory (Steneck 1988; Fox and Bellwood 2007), all methods were also replicated along four distinct depth contours (2, 5, 8, 12-m depth) at individual sites with similar reef profiles. From these data, we tested the overarching null hypothesis that overall relative herbivory would not differ between thermal realms. Additionally, we tested the following sub-hypotheses: 1) relative fish herbivory would be highest on tropical reefs and decrease with depth, 2) relative benthic herbivory would exceed fish herbivory on temperate reefs and decrease with depth, and 3) nominally herbivorous fish abundance and biomass would be higher on tropical reefs, whereas urchin abundance and biomass would be higher on temperate reefs.

## Materials and methods

### Study sites

**Fig. 1:**
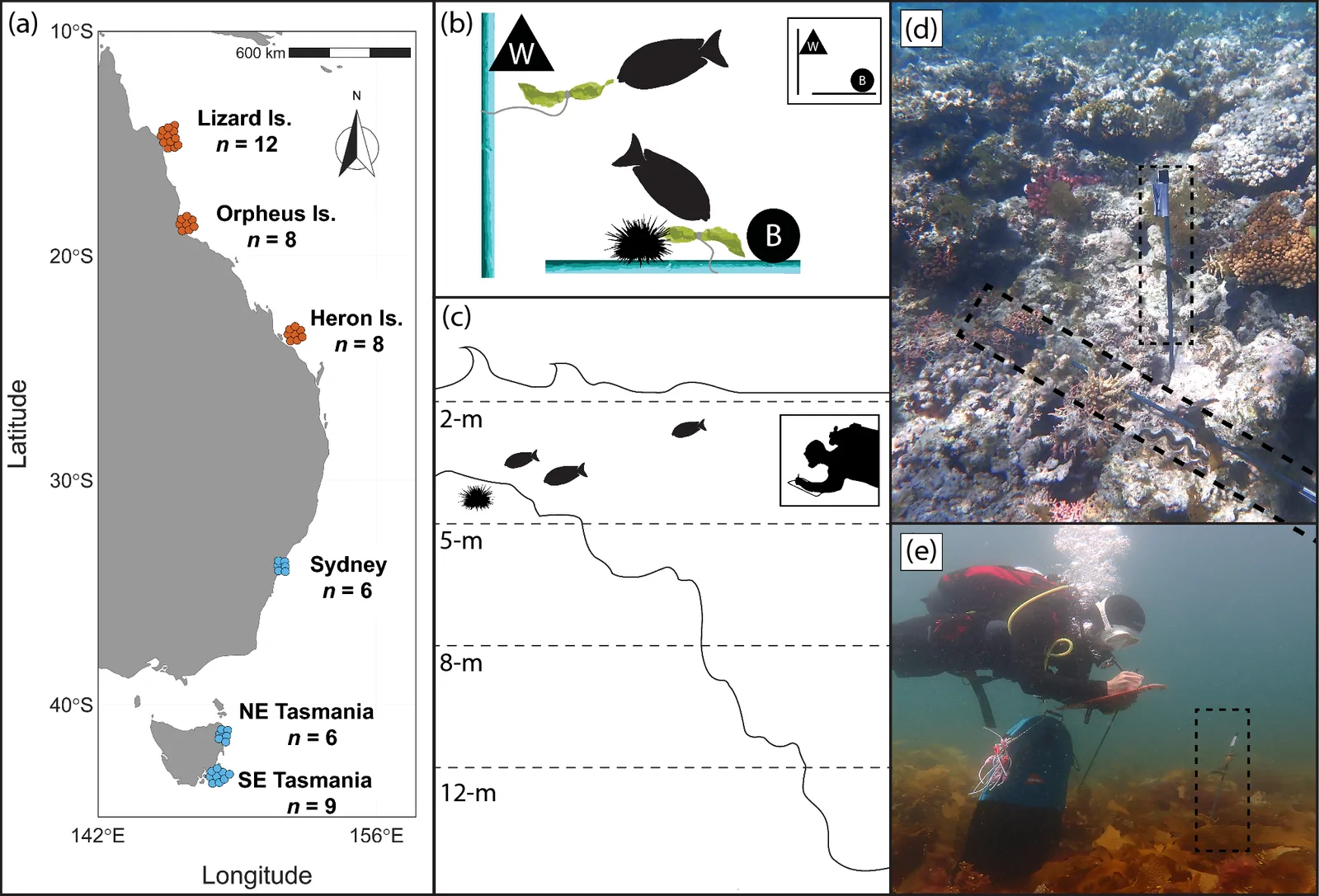
Tropical (orange) and temperate (blue) study locations spanning approximately 29° of latitude and schematic of experimental design including assays of herbivory and underwater visual surveys of herbivores. (**a**) Map of eastern Australia showing experimental sites (coloured circles jittered for clarity) with n = number of total sites within each of the three tropical and three temperate locations (N = 49). (**b**) Schematic of assay treatments partitioning consumption of the standard alga *Ulva lactuca* by fishes in the water-column (‘W’ depicted within triangle) and herbivory by fishes and urchins on the benthos (‘B’ in circle), noting images are not drawn to scale. (**c**) Schematic of depth-stratified design of herbivory assays and herbivore surveys at four depth contours (2, 5, 8, and 12-m) which was performed at a single site within each of the six locations. Example photos captured in situ are shown for (**d**) tropical assays on a coral reef and (**e**) temperate assays in a kelp bed, with black bounding boxes highlighting water-column and benthic assays

Fieldwork was undertaken from 2022–2025 at three tropical and three temperate locations in eastern Australia. To examine overall thermal realm effects, experimental assays and surveys were conducted in the shallow subtidal zone at 21 temperate reef sites and 28 tropical reef sites (2—12-m depth). Tropical sites were located at Lizard (n = 12), Orpheus (n = 8), and Heron (n = 8) Islands (Fig. 1a), which were characterised by a mosaic of live scleractinian corals, coral rubble, and turf algae across depths. Temperate sites were located in Sydney (n = 6), NE Tasmania (n = 6), and SE Tasmania (n = 9), which were dominated by the laminarian kelp *Ecklonia radiata*, interspersed with turf algae, sponges, and open reef patches (Fig. 1a; see Online Resource 1 key site features and deployment details). To categorically examine the effects of thermal realm and depth, these methods were further replicated along the 2, 5, 8, and 12-m depth contours at three select sites within each of the tropical and temperate reef locations which had similar depth gradients. Mean water temperature at the time of deploying herbivory assays was 25.1 °C ± 0.45 SE for tropical sites and 17.6 °C ± 0.62 for temperate sites, as recorded in situ with a dive computer.

### Quantifying herbivory

To estimate the relative intensity of herbivory, we deployed standardised macroalgal assays (following Ling et al. 2018; Tebbett et al. 2025b; Rose et al. 2026). To control for latitudinal variation in algal defences, we purchased a standardised dehydrated *Ulva lactuca* product, i.e., ‘sea lettuce’, sold for human consumption. *Ulva lactuca* has previously been used to quantify herbivory and is readily consumed by both fish and urchins (Taylor and Steinberg 2005; Ling et al. 2018; Della Marta et al. 2023; Rose et al. 2026). *Ulva* was rehydrated immediately before deployment then cut into 20 by 70 mm pieces. Algal pieces were attached to 150 mm lengths of 8 kg breaking strain fishing line using cable-ties (140 mm by 3.5 mm), with each length of fishing line tied to 650 mm long fibreglass garden stakes (Fig. 1b, c, d). Every assay deployment comprised 10 assays suspended in the water-column that only fish could consume (water-column herbivory), and 10 assay stakes lying flat on the benthos that fish and urchins could both consume (benthic herbivory) (n = 20 assays per deployment). To robustly test thermal realm-level effects, assay deployments were replicated along one depth contour at 28 tropical and 21 temperate sites, constraining deployments to one contour to maximise site replication. Then, for controlled testing of depth effects, a single site within each location was selected (n = 6), to further replicate assay deployments and visual surveys at the 2, 5, 8, 12-m depth contours, providing two data sets for separate analyses (See Online Resource 1 for overview of single-depth sites and cross-depth sites).

Replicate pairs of each assay partitioning treatment were placed at 5-m intervals along a 50-m transect. Due to natural variation in benthic composition, assays were deployed in a variety of ways, but urchins were always unable to consume water-column assays. Primarily, stakes were wedged securely into crevices or boulder structures formed by rock or dead coral. For water-column assays, if an appropriate crevice was not available, fine gauge 4 mm diameter bungee clips were used to secure stakes to local vertical structures such as macroalgal stipes or dead coral branches. If a macroalgal stipe was used to suspend an assay in the water-column, fronds were removed to ensure assay offerings projected above the canopy. All assays were deployed at haphazard times during the day and remained in situ for approximately 24-h to encompass periods of diurnal variation in fish herbivory and nocturnal peaks in urchin activity (deployment times; Online Resource 1). Upon retrieval, each algal piece was examined and scored for percent consumption as 0, 25, 50, 75, or 100%. Additionally, these scores were also converted to a binary response of 1 (consumed [i.e. any level of consumption]) or 0 (not consumed [i.e. nil observed consumption]), to allow separate analyses on percent and binary consumption responses. No control treatment was included, but 71.5% of all assays remained completely intact and present, suggesting that physical dislodgement did not occur systematically, and fish were frequently observed consuming assay material. While some assay material may be lost to non-herbivory sources, direct feeding observations across multiple studies and high retention of assay material suggests that physical degradation does not confound relative herbivory patterns (Ling et al. 2018; Tebbett et al. 2025b; Rose et al. 2026).

### Visual surveys of herbivore communities

We determined the relative abundance and biomass composition of fish and urchin species, through standardised visual surveys at each site (and for each depth contour at explicit depth-stratified sites) following the Reef Life Survey method (Edgar et al. 2020). Reef fish and urchins were observed by two divers along a pre-laid 50-m transect line deployed at a uniform depth contour. Observations of fish were made within 5-m wide ‘blocks’ either side of the transect line (250-m^2^ each), while subsequent urchin observations were made within 1-m blocks (50-m^2^ each). Herbivores were identified to the species level, their abundance recorded, and allocated to the nearest size category of 2.5, 5, 7.5, 10, 12.5, 15, 20, 25, 30, 35, 40, and 50-cm in total length, with rounding to nearest 12.5-cm above 50-cm (Edgar et al. 2020). Size was estimated as total length for fish, from their snout to the apex of their caudal fin, and as test diameter for urchins. Abundance was converted to biomass estimates using length–weight regression parameters: fish parameters were sourced from FishBase (Froese et al. 2014; Froese and Pauly 2025), and published class-level parameters were used for urchins (Heather et al. 2021). All biomass estimates were standardised to 1-m^2^ and averaged across survey blocks, to account for the non-independence of blocks arising from their spatial proximity. For subsequent analyses of herbivore abundance and biomass, we included select groups of nominal herbivores likely to consume assays and excluded those with feeding traits that made them more unlikely to target assays. From tropical survey data, we excluded Acanthurid ‘brushers’ and ‘sediment suckers’ as they target detritus, have a limited capacity to consume algae and/or predominantly feed over sand (Tebbett et al. 2017, 2025a), and the sea urchin *Echinostrephus* spp., as they are sedentary burrowing species that predominantly feed on drifting material (Asgaard and Bromley 2008) (for species list, see Online Resource 2).

### Erect macroalgae cover

For sites used to test depth effects, photographs of habitat composition were taken at each assay position along each transect (n = 10 per depth per site; see example images Online Resource 3), centred approximately 1-m above the paired assays (equating to an approximate area of 1.5-m^2^). Percent cover of erect macroalgae was then scored from recorded images by overlaying 100 equidistant points and tallying the total points intercepting macroalgae.

### Statistical analyses

All analyses used generalised linear mixed effects models (GLMMs), with fitting and checking procedures undertaken in R (R Core Team 2025), using the *car* (Fox and Weisberg 2019), *glmmTMB* (Brooks et al. 2017), *emmeans* (Lenth 2020), and *DHARMa* (Hartig 2020) packages. Candidate model comparisons were conducted using corrected Akaike Information Criterion values (AICc) and the model with the lowest AICc was selected to make inferences (in cases where models were within 2 AICc units they were considered equivalent and the simpler model was used) (AICc summary tables; Online Resource 4). Selected models were validated using diagnostic plots based on simulated residuals, all of which suggested model fit was satisfactory and assumptions were met. Type II analysis of deviance (ANOVA) was used to assess the overall significance of fixed effects, and Tukey-adjusted pairwise comparisons were undertaken for post-hoc analyses (summaries of statistical outputs Online Resources 5–7). Firstly, overall thermal realm effects on assay consumption, herbivore abundance, and herbivore biomass were examined using the broader suite of sites encompassing 21 temperate and 28 tropical sites (inclusive of the 5-m depth data from the depth-stratified sites). Secondly, the effects of thermal realm and depth were examined in combination using the cross-depth data from sites where depth was explicitly tested by categorically replicating assay deployments and visual surveys at the 2, 5, 8, and 12-m depth contours.

Across all herbivory data, 71.5% of assays were untouched and 62.7% of consumed assays were completely consumed within the 24-h deployment time. Therefore, herbivory was analysed as a binary response (1 = consumed [inclusive of any level of consumption], 0 = not consumed [i.e. untouched]) using a binomial GLMM and logit link function, fitted with restricted maximum likelihood to best handle imbalance in the number of sites per location which varied across thermal realm. To solely test the effect of thermal realm on consumption, a binomial GLMM was fitted with thermal realm and partitioning as fixed effects in a two-way interaction, with hierarchically nested random effects included for location, site and replicate stake pair identity. When testing thermal realm and depth effects in combination, thermal realm, depth, and partitioning were fitted as fixed effects, and replicate stake pair identity nested in site included as a random effect. AICc selected the two-way interaction structure: thermal realm × depth + thermal realm × partitioning. Additionally, herbivory patterns were analysed from percent-consumed data (0, 25, 50, 75, 100%) fitted using a beta-binomial GLMM and logit link function, with identical fixed and random effects structure for the cross-depth data. Ecological interpretations did not differ between models and only the binary model is presented in the main text (for beta-binomial outputs see Online Resource 4b, 5c, 6c, 7c).

Survey data were divided into fish and sea urchin abundance and biomass for separate analyses, each fitted as a Tweedie GLMM using a log-link function. Again, the overall effect of thermal realm was tested by fitting thermal realm as a singular fixed effect, with location included as a random effect. In this context, the purpose of the GLMMs based on survey data were to explore distribution patterns in nominally herbivorous fishes and urchins across thermal realms and depth, rather than make quantitative links between community metrics and relative consumption patterns. When testing thermal realm and depth effects in combination, AICc selected the fixed effects structure thermal realm + depth in all cases, with location included as a random effect in all four models. Lastly, photographs at each assay position (example images; Online Resource 3) showed that erect algae cover was almost entirely absent from tropical replicates, with numerous factor levels composed entirely of zeroes, prohibiting reliable model fit. Therefore, data for the proportional cover of erect macroalgae are only presented graphically.

## Results

### Macroalgal assays

**Fig. 2:**
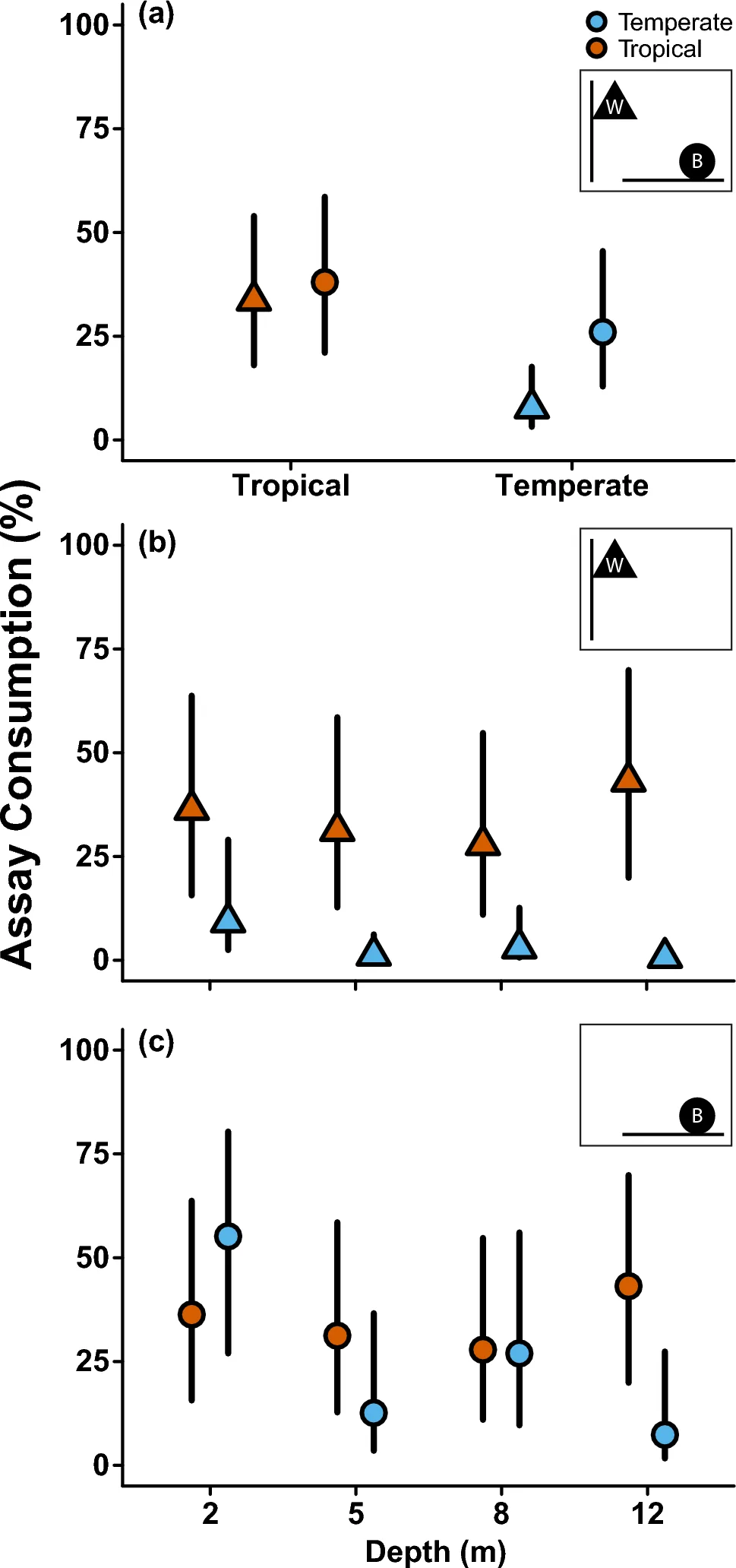
Percent consumption of algal assays suspended in the water-column (triangles) and laying across the benthos (circles) consumed between tropical (orange) and temperate (blue) thermal realms. (**a**) water-column and benthic assay consumption by thermal realm, (**b**) water-column assay consumption measuring relative fish herbivory by thermal realm and depth (2, 5, 8 and 12-m), and (**c**) benthic assay consumption by thermal realm and depth. For (a), assay partitioning differed significantly for temperate reefs but not tropical (temperate realm × water-column, *P* <0.001). For realm-level patterns in (a), models were conducted using data across n = 28 tropical and n = 21 temperate single-contour sites (N = 49); (b) and (c) were conducted using data from the six depth-stratified sites (one site per location, N = 6). Large points and line ranges denote estimated marginal means with 95% confidence intervals from generalised linear mixed effects models

In partitioning relative algal consumption in the water-column (fish herbivory) and on the benthos (fish and urchins) across sites, there were significant interactive effects for thermal realm and partitioning treatment (*P* <0.001; Fig. 2; see GLMM summaries Online Resource 5a). Between the two thermal realms, fish consumption was higher on tropical reefs (*P* = 0.026), whereas benthic consumption did not differ (*P* = 0.80; Fig. 2; see pairwise comparisons Online Resource 7a). Moreover, both assay types were consumed equally on tropical reefs (*P* = 0.76), while on temperate reefs benthic consumption was higher than fish assay consumption (*P* <0.001; Fig. 2a; see pairwise comparisons Online Resource 7a).

In partitioning algal consumption between the water-column and the benthos across depth-stratified sites, there were significant interactive effects for thermal realm and depth (*P* = 0.001) as well as thermal realm and partitioning treatments (*P* <0.001; Fig. 2; see GLMM summaries Online Resource 5b, c). As for the full set of sites above, fish consumption was higher on tropical reefs compared to temperate reefs (*P* = 0.003), whereas benthic consumption did not differ between tropical and temperate reefs (*P* = 0.82; Fig. 2b, c; see GLMM summaries Online Resource 5b, c). Additionally, when combining fish and benthic herbivory for the depth-stratified experiment, tropical assay consumption was higher than temperate assay consumption at 5-m and 12-m depth (*P* <0.01 in both cases; Fig. 2b, c; see pairwise comparisons Online Resource 7b, c). On tropical reefs, fish herbivory and benthic herbivory did not differ at any depth (Fig. 2b, c). Additionally, temperate herbivory overall decreased across depth, with consumption odds being 2.1 ± 0.62 SE times lower at 5-m depth and 2.7 ± 0.72 lower at 12-m depth relative to 2-m depth (*P* <0.05 in both cases; Fig. 2b, c; see pairwise comparisons Online Resource 7b, c).

### Herbivore abundance and biomass

**Fig. 3:**
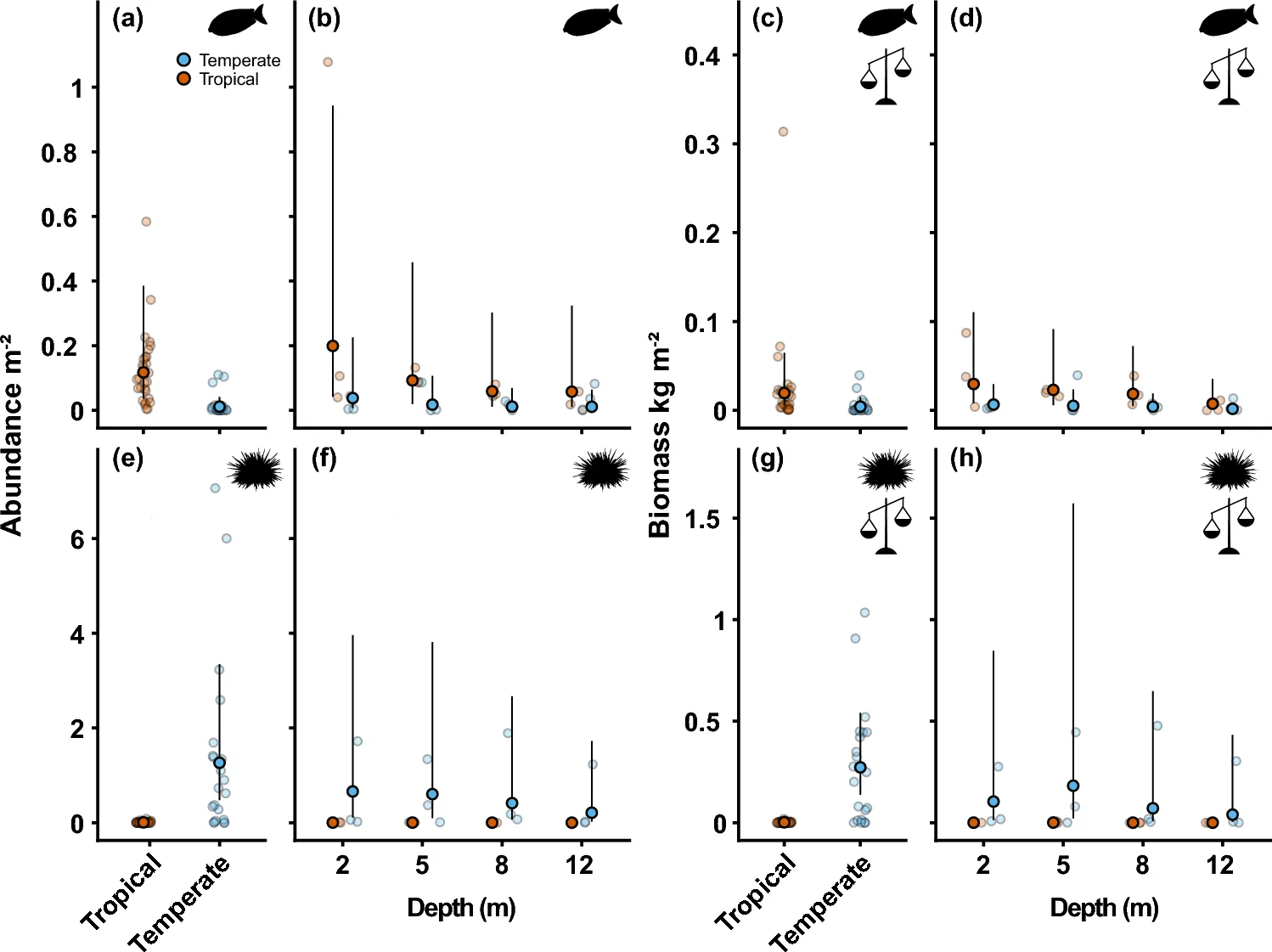
Surveys of nominal herbivore groups by thermal realm and depth. Herbivore abundance (m^−2^) and biomass (kg m^−2^) by depth on tropical (orange) and temperate (blue) reefs for fish (top panels: a-d) and urchins (bottom panels: e–h). Large points and line ranges show estimated marginal means with 95% confidence intervals from generalised linear mixed effects models, with small translucent points denoting raw survey observations which are jittered for clarity, (n = 49 surveys for (a), (c), (e), (g); n = 24 surveys for (b), (d), (f), (h); inclusive of the 5-m depth data from the depth-stratified sites). Black icons of herbivore groups denote model responses; an individual fish or urchin for abundance models and an individual with a set of scales for biomass

Visual survey estimates for the abundance and biomass of nominally herbivorous fish and urchins revealed a switch in dominance from fish to urchins between tropical and temperate reefs (Fig. 3a, c, e, g; see ANOVA results Online Resource 5d, e, h, i). When examining the combined effects of thermal realm and depth, the abundance and biomass of urchins remained significantly higher on temperate reefs compared to tropical reefs, whereas surveyed fish abundance and biomass did not differ (Fig. 3b, d, f, h; see ANOVA results Online Resource 5f, g, j, k). Indeed, average urchin abundance was 145-fold higher on temperate reefs compared to tropical reefs and average urchin biomass was 141-fold higher. Across depths, there was no significant variation in the surveyed abundance and biomass of fish or urchins in either thermal realm (Fig. 3b, d, f, h; see ANOVA results Online Resource 5f, g, j, k).

### Erect macroalgal cover

**Fig. 4:**
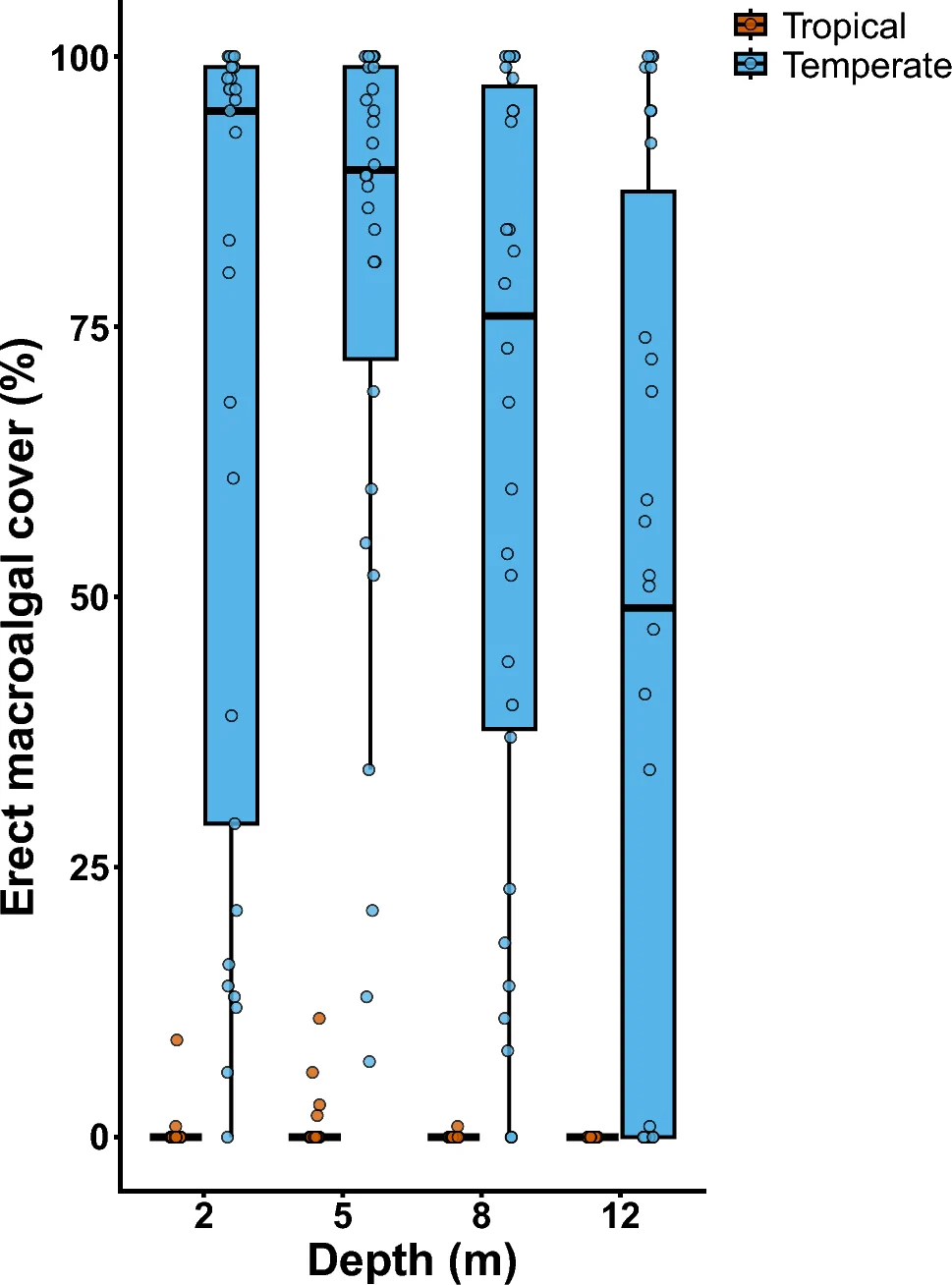
Percent cover of erect macroalgae at four depths (2, 5, 8, and 12-m) in tropical (orange) and temperate (blue) thermal realms (n = 3 sites per thermal realm). Boxplots represent interquartile range (IQR) including median and first (Q1) and third (Q3) quartiles (25th and 75th percentiles), minimum whisker calculated as: Q1–1.5 × IQR, maximum calculated as: Q3 + 1.5 × IQR. Small translucent points denote raw percent values derived from photo-quadrats captured at each assay position which are jittered for clarity (N = 240)

A distinct pattern in erect macroalgal cover was evident between thermal realms, with an average 213-fold higher overall cover on temperate reefs (Fig. 4). Erect macroalgal cover was virtually absent at tropical locations, with an average cover of 0.3% ± 0.1 SE; the heavily calcified *Halimeda* sp. comprised all erect tropical algae. Mean macroalgal cover on temperate reefs was 64.1% ± 3.5, with *Ecklonia radiata*, *Phyllospora comosa*, and *Sargassum* sp. dominating temperate algal composition, in descending rank order. Average temperate macroalgal cover exhibited a general negative relationship with depth, with cover peaking at 79% ± 5.1 at 5-m depth and declining to 44.6% ± 7.5 at 12-m depth.

## Discussion

### Herbivory patterns between thermal realms

Studies on reef fish herbivory have generally shown marked decreases in nominal herbivore abundances and herbivory of benthic macrophytes with increasing distance from the equator (e.g., Gaines and Lubchenco 1982; Longo et al. 2019; Tebbett et al. 2025c). Here, when considering only water-column assays, consumption patterns supported this general pattern of a decline in relative fish herbivory with increasing latitude (Fig. 2a). However, when considering the consumption of both assay partitioning treatments in combination, results indicated no difference in overall relative herbivory levels between thermal realms (Fig. 2a, b). Although, consumption patterns indicated a dominance of benthic herbivory on temperate reefs. Collectively, our assays and surveys indicate that urchins were the primary benthic herbivore driving this signal, as they were the most frequently observed consumer and are well known to exert high herbivory pressure across our temperate region (Ling and Keane 2024; Rose et al. 2026). However, notable exceptions have been documented elsewhere, with fish herbivory playing an important role in structuring macroalgae diversity and distribution on temperate reefs in these cases (e.g., Harris et al. 1984; Clements and Choat 1993; Ojeda and Muñoz 1999). Partitioning fish and urchin herbivory at our tropical sites was more difficult because both partitioning treatments were consumed relatively frequently and while tropical urchins were relatively rare, their abundance can be underestimated from daytime visual surveys alone (Jones et al. 2026).

### Constrained *versus* diffuse herbivory

Temperate herbivory was spatially constrained by occurring primarily on the benthos, whereas tropical herbivory occurred in a diffuse manner, more evenly across both the water-column and the benthos. In comparison to fish, urchin herbivory is also highly concentrated (Eynaud et al. 2016), with the movement and feeding of urchins heavily restricted to their immediate environment (Jones and Andrew 1990). Indeed, for the dominant urchin across our temperate study regions, *Centrostephanus rodgersii,* their herbivory is well predicted by their local abundances (Rose et al. 2026), as they move <10-m per night and often return to the same individual crevice (Flukes et al. 2012; Ling et al. 2016). In contrast, free-swimming fish rove over reef scapes at scales of tens to thousands of metres in search of food (Ogden and Lobel 1978), with some herbivorous fish travelling up to 11-km a day and thus distributing their feeding across relatively greater distances (Welsh and Bellwood 2014; Eynaud et al. 2016; Streit et al. 2019).

Ultimately, differences in how and where herbivory occurs, alongside interactions with reef topography, appear to shape macroalgae distribution on temperate reefs. For example, high-relief crevice structures can promote urchin populations, creating positive feedback between rugosity and herbivory within the benthic plane (Andrew 1993; Byrne and Andrew 2020). This can be observed across our temperate study locations as the incipient barrens patches which ultimately ensue from increased *C. rodgersii* populations, radiate outward from crevices before coalescing and forming more extensive urchin barrens (Flukes et al. 2012). In contrast, elevations in diffuse fish herbivory, resulting from tropicalization, can underpin an opposite effect, restricting macroalgae distribution to within crevice structures that provide refuge from herbivorous fish targeting exposed upright macroalgae (Bennett et al. 2010; Zarco‐Perello et al. 2021). These examples highlight that the dominant plane of herbivory within a given reef system can have important effects on where macroalgae can persist.

### Top-down predation shapes herbivory

On temperate reefs, a dominance of benthic herbivory emerged and coincided with higher urchin biomass, a pattern that may arise from latitudinal variation in predation pressure. Community structure is heavily influenced by top-down predation pressure, with predators shaping herbivore composition and herbivory over evolutionary and ecological timescales (Estes et al. 2011; Steneck 2020). Indeed, similarly designed predation assays have demonstrated latitudinal shifts in relative predation, alongside shifts from fish to crab-dominated predation (Duffy et al. 2015; Musrri et al. 2019, 2026; Muething et al. 2020). Such generality in shifts from vertebrate to invertebrate dominance across thermal realms suggests that overarching mechanisms govern vertebrate and invertebrate dominance (Musrri et al. 2019), irrespective of trophic group. This shared pattern may have a consistent temperature-mediated physiological basis, whereby fishes are more constrained by cooler waters via impacts on their metabolism and digestion, imposing latitudinal thresholds on fish dominance that, in turn, promotes invertebrate dominance at higher latitudes (Behrens and Lafferty 2007; Iken et al. 2010). These patterns may be increasingly disrupted as oceans continue to warm.

When trophic structure is disrupted, release from predation pressure can cause trophic cascades that redefine herbivore dominance and herbivory (Estes et al. 2011; Ripple and Beschta 2012; Ling et al. 2015). For example, when predation on sea urchins decreased on Caribbean reefs from overfishing of key predators, the sea urchin *Diadema antillarum* rose to prominence as a pivotal herbivore (Hay 1984; Hughes 1994; Mumby et al. 2006). The influence of their herbivory was well demonstrated following mass die-offs and subsequent increase in macroalgae (Lessios 2016). On temperate reefs, reduced predation pressure and competition from fewer herbivorous and carnivorous fish may allow urchins to dominate herbivory patterns (Meekan and Choat 1997; Floeter et al. 2005; Steneck 2020). Notably, urchin predators that do inhabit temperate reefs in our study region have been heavily fished (Ling et al. 2009; Colley and Attenbrow 2012; Ling and Johnson 2012; Young et al. 2014). As such, our assay results may partly reflect a disruption to top-down predation pressure, as has been documented on Caribbean coral reefs and elsewhere.

### Depth gradients and foraging behaviour

Variation in both the distribution of herbivores (Steneck 1988; Scott et al. 2022; Pinheiro et al. 2023) and in the intensity of herbivory rates (Hay 1981; Morrison 1988; Fox and Bellwood 2007) across depth gradients has been widely reported. On temperate reefs, a negative trend in benthic herbivory with increasing depth was evident, with highest consumption at 2-m depth (Fig. 2c). A declining trend in herbivory with increasing water depth is likely linked, at least in part, to variation in key environmental factors such as light intensity and wave energy that vary strongly across this gradient and structure herbivore communities both directly and indirectly (Copertino et al. 2005; Fox and Bellwood 2007; Bejarano et al. 2017; Laverick et al. 2020). For example, algal productivity is often maximised in shallow reef areas, with herbivore populations known to track productivity on reefs (Russ 2003; Tootell and Steele 2016; Kriegisch et al. 2020). For tropical fish herbivory in the current study, the lack of a depth effect may partly reflect our small depth gradient (2–12-m), especially given that previous studies examining herbivory to depths of 30-m (Hay 1981) and 40-m (Morrison 1988), report strong depth patterns in tropical herbivory. Moreover, the high variance and relatively small sample size likely constrained our capacity to detect an effect in this case, especially in terms of the survey data. In this respect, herbivorous fish can rove over large distances (Welsh and Bellwood 2014, 2015), and the methods, including the relatively small area surveyed, may have resulted in lower detectability during visual surveys limiting our capacity to detect differences (also see Jones et al. 2025). As such, our cross-depth sampling could be extended across a greater depth range at a broader range of sites and include greater within-site replication to better represent herbivorous fish communities in future research.

### Reef states and tropicalization signals

Surveys revealed virtually no cover of erect macroalgae on tropical reefs, but high erect macroalgal cover on temperate reefs, representing the states that typically characterise each thermal realm (Gaines and Lubchenco 1982; Cresswell et al. 2017). Notably, the dominance of macroalgae at temperate sites may have influenced the response of temperate fish herbivory to our assay method. These same assays have detected higher temperate fish herbivory on turf-dominated reefs across the same temperate study regions, alongside higher urchin herbivory (Rose et al. 2026). Indeed, habitat context (i.e. macroalgae vs turf dominated) can produce bottom-up feedback loops that likely modify how herbivory occurs (Rose et al. 2026). As oceans warm, the loss of erect macroalgae and formation of turf-dominated temperate reefs is being increasingly documented, a benthic transition that is often linked to elevated herbivory (Vergés et al. 2014; Filbee-Dexter and Wernberg 2018; Ruiz-Ruiz et al. 2025). Due to strong habitat selectivity by species, many range-extending taxa preferentially establish on turf-dominated tropicalized reefs (Stuart-Smith et al. 2021; Schuster et al. 2022). Therefore, it is likely that shifts from macroalgae to turf dominance may produce positive feedback loops that promote herbivorous fish establishment and further herbivory (e.g., Bennett et al. 2015). Moreover, other studies suggest it may not simply be increased feeding but also changes to how and where herbivory occurs, that impact algal distribution patterns on temperate reefs (Zarco‐Perello et al., 2020, 2021). In the current study, patterns in temperate herbivory between the water-column and benthos indicate that the functional space of temperate herbivory likely represents a key axis of variation in a warming world (Ripple et al. 2023). As herbivorous fish progressively invade temperate reefs through tropicalization, (Vergés et al. 2014, 2016; Bennett et al. 2015; Inagaki et al. 2020; Zarco‐Perello et al. 2020; Soler et al. 2022), herbivory is likely to become less spatially constrained and concentrated. Moreover, increasing temperatures may progressively elevate herbivore metabolisms (Gutow et al. 2016), collectively increasing the intensity of herbivory on both standing and low-lying algae by a variety of fish functional groups. However, the realised impact of such shifts is likely to be mediated by the palatability of algae that become less chemically defended at higher latitudes, along with the dietary preferences and metabolic demands of redistributing species (Taylor and Steinberg 2005; Musrri et al. 2026).

## Conclusions

Our macroalgal assay results indicated that relative herbivory levels were similar between tropical and temperate reefs. While both assay partitioning treatments were consumed equally on tropical reefs, a strong partitioning towards benthic herbivory, attributable to urchins, was evident on temperate reefs. These patterns of relative herbivory will likely be reshaped if ocean warming and tropicalization support further increases in the relative dominance of fish compared to urchins on temperate reefs. A tropicalized herbivory regime, whereby novel fish and urchin herbivory increases, would likely lead to amplified herbivory with maximal reductions in standing algal cover. Alternatively, shifts in community structure and potential for competitive exclusion between herbivores may only allow either fish or urchins to dominate. Ultimately, shifts in where and how herbivory occur appears central to the process of tropicalization and its impact on macroalgal dominance on temperate reefs.

## Supplementary Information

Below is the link to the electronic supplementary material.

Supplementary file 1.

Supplementary file 2.

Supplementary file 3.

Supplementary file 4.

## Data Availability

The data are provided as a supplementary excel file.

## Code availability

The analysis code is provided as a supplementary R file.

## Declarations

### Highlighted student paper

*This paper deserves recognition as a Highlighted Student Paper given the spatial extent of the research and novel documentation of patterns in fish and urchin herbivory across tropical and temperate reefs.*
