# Supplementary material for: Partitioning patterns of fish and sea urchin herbivory from tropical to temperate reefs
Source: Oecologia. 2026 Oct 25;208(11):156. doi: 10.1007/s00442-026-05997-0 (PMC13696572; doi:10.1007/s00442-026-05997-0)
Supplement: Supplementary file 1 [file 442_2026_5997_MOESM1_ESM.pdf]

Electronic Supplementary Materials

Partitioning patterns of fish and sea urchin herbivory from tropical to temperate reefs

Matthew Rose*, Paula A. Ruiz-Ruiz, Sterling B. Tebbett, Tyson R. Jones, Yann Herrera-Fuchs, Lara Denis-Roy, Claire Butler, Scott D. Ling

Affiliation

Institute for Marine and Antarctic Studies, University of Tasmania, Hobart, Tasmania, Australia 7004

Online Resource 1 Tabulated site details showing realm, site name, dominant biogenic habitat-former, depth, latitude and longitude, date of deployment, deployment time, and temperature at the time of deployment for each site within each of the three tropical and temperate locations, spanning approximately 29° of latitude. a) Single depth contour sites within each location, and b) sites within each location where herbivory assays were replicated across depths contours (2, 5, 8, 12 m depth).

Online Resource 1a - Single depth contour sites

Realm	Location	Site	Habitat	Lat	Lon	Date	°C	Depth	Deployment
Tropical	Heron Is.	Coral Gardens	Mixed Corals	-23.4472	151.9120	31/05/2022	24	8	03:30:00 PM
Tropical	Heron Is.	East Yellow	Mixed Corals	-23.4337	151.9645	03/06/2022	23	5.5	04:00:00 PM
Tropical	Heron Is.	Fourth Point	Mixed Corals	-23.4373	151.9813	03/06/2022	23	10	02:35:00 PM
Tropical	Heron Is.	Heron Bommie	Mixed Corals	-23.4416	151.9050	30/05/2022	24	6	09:30:00 AM
Tropical	Heron Is.	Russdive Wistari	Mixed Corals	-23.4661	151.8507	02/06/2022	23	7.2	01:00:00 PM
Tropical	Heron Is.	Tenements 2	Mixed Corals	-23.4333	151.9317	01/06/2022	24	8	12:00:00 PM
Tropical	Heron Is.	Westari	Mixed Corals	-23.4607	151.8633	03/06/2022	23	7.3	11:00:00 AM
Tropical	Lizard Is.	Bird Islet	Mixed Corals	-14.6914	145.4678	19/05/2023	27	4	10:30:00 AM
Tropical	Lizard Is.	Bird Islet Crest	Mixed Corals	-14.6914	145.4678	19/05/2023	27	2	12:00:00 PM
Tropical	Lizard Is.	Blue Hole	Mixed Corals	-14.6947	145.4592	19/05/2023	27	3	01:30:00 PM
Tropical	Lizard Is.	Casuarina	Mixed Corals	-14.6775	145.4435	27/05/2023	27	3.5	02:30:00 PM
Tropical	Lizard Is.	Horseshoe Reef	Mixed Corals	-14.6871	145.4433	28/05/2023	27	3	11:00:00 AM
Tropical	Lizard Is.	Loomis Reef	Mixed Corals	-14.6829	145.4491	27/05/2023	27	2	09:00:00 AM
Tropical	Lizard Is.	Osprey	Mixed Corals	-14.6654	145.4423	26/05/2023	27	4	01:00:00 PM
Tropical	Lizard Is.	Palfrey South	Mixed Corals	-14.6967	145.4477	28/05/2023	27	5	12:00:00 PM
Tropical	Lizard Is.	Turtle Reef	Mixed Corals	-14.6553	145.4505	26/05/2023	27	5	11:00:00 AM
Tropical	Lizard Is.	Vickys Reef	Mixed Corals	-14.6869	145.4420	22/05/2023	27	6	02:15:00 PM
Tropical	Lizard Is.	Watsons Bay north	Mixed Corals	-14.6571	145.4508	19/05/2023	27	5	09:00:00 AM
Tropical	Orpheus	East Pelorus	Mixed Corals	-18.5610	146.5014	30/01/2025	29	9	09:30:00 AM
Tropical	Orpheus	Iris Point	Mixed Corals	-18.5680	146.4821	02/08/2024	22	7	10:30:00 AM
Tropical	Orpheus	Little Pioneer Bay	Mixed Corals	-18.5931	146.4832	03/08/2024	22	4.5	10:30:00 AM
Tropical	Orpheus	NE Orpheus	Mixed Corals	-18.5899	146.4981	30/01/2025	29	9	09:30:00 AM
Tropical	Orpheus	North Pioneer Bay	Mixed Corals	-18.6059	146.4886	03/08/2024	22	6	10:30:00 AM
Tropical	Orpheus	Snapper Pt	Mixed Corals	-18.6599	146.5118	30/01/2025	29	10	09:30:00 AM
Tropical	Orpheus	Southwest Pelorus	Mixed Corals	-18.5546	146.4880	01/08/2024	22	7.5	10:30:00 AM
Temperate	NE Tas	Bicheno Gulch	*Ecklonia radiata*	-41.8698	148.3117	28/03/2024	17.5	10	12:00:00 PM
Temperate	NE Tas	Elephant Rock	*Ecklonia radiata*	-41.2537	148.3397	05/05/2024	16.5	12	12:00:00 PM
Temperate	NE Tas	Pegg's Point	*Ecklonia radiata*	-41.8680	148.3043	28/03/2024	17.5	10	10:00:00 AM
Temperate	NE Tas	Sloop Rock	*Ecklonia radiata*	-41.2103	148.2934	05/05/2024	16.5	12	10:00:00 AM
Temperate	NE Tas	St. Helens Point	*Ecklonia radiata*	-41.2735	148.3545	05/05/2024	16.5	9	03:00:00 PM
Temperate	SE Tas	Blackmans Bay	*Ecklonia radiata*	-43.0075	147.3281	23/01/2023	17.7	4.5	03:00:00 PM
Temperate	SE Tas	Crayfish Point	*Ecklonia radiata*	-42.5711	147.2116	04/01/2023	16.5	3.5	04:00:00 PM
Temperate	SE Tas	Isle of The Dead	*Ecklonia radiata*	-43.1485	147.8694	28/03/2024	17	8.5	04:00:00 PM
Temperate	SE Tas	Lookout Bay	*Ecklonia radiata*	-43.1707	147.4077	13/04/2024	17	10	12:00:00 PM

Temperate	SE Tas	Penguin Tiles	*Ecklonia radiata*	-43.3488	147.3723	02/06/2024	13	9	02:30:00 PM
Temperate	SE Tas	Primrose Sands	*Ecklonia radiata*	-42.8981	147.6690	06/04/2023	13	4	02:00:00 PM
Temperate	SE Tas	Safety Cove	*Ecklonia radiata*	-43.1845	147.8698	28/03/2024	17	8	02:00:00 PM
Temperate	SE Tas	Trumpeter Bay	*Ecklonia radiata*	-43.1644	147.3979	13/04/2024	17	10	09:00:00 AM
Temperate	Sydney	Camp Cove	*Ecklonia radiata*	-33.8401	151.2775	27/11/2024	22	5	10:00:00 AM
Temperate	Sydney	Chowder North	*Ecklonia radiata*	-33.8380	151.2616	28/11/2024	22	5	10:00:00 AM
Temperate	Sydney	Chowder South	*Ecklonia radiata*	-33.8446	151.2534	28/11/2024	22	5	12:00:00 PM
Temperate	Sydney	Middle Head	*Ecklonia radiata*	-33.8288	151.2653	26/11/2024	22	5	09:00:00 AM
Temperate	Sydney	Shark Point	*Ecklonia radiata*	-33.8484	151.2680	28/11/2024	22	5	02:00:00 PM
Temperate	SE Tas	Primrose Sands	*Ecklonia radiata*	-42.8981	147.6690	06/04/2023	13	4	2:00:00 PM

Online Resource 1b - depth replicated sites

Realm	Location	Site	Habitat	Lat	Lon	Date	°C	Depth	Deployment
Tropical	Lizard Is.	Mermaid Cove	Mixed corals	-14.6461	145.4539	21/05/2023	27	2	12:30:00 PM
								5	11:00:00 AM
								8	12:30:00 PM
								12	11:00:00 AM
Tropical	Orpheus Is.	Pioneer Bay	Mixed corals	-18.6125	146.4827	16/07/2024	29	2	7:00:00 PM
								4	7:00:00 PM
								8	8:00:00 AM
								12	8:00:00 AM
Tropical	Heron	Wistari Reef	Mixed corals	-23.4542	151.9108	11/02/2023	21	2	10:00:00 AM
								5	1:15:00 PM
								8	1:15:00 PM
						07/02/2023		12	10:30:00 AM
Temperate	Sydney	South Head	*Ecklonia radiata*	-33.8330	151.2800	29/08/2024	17	2	9:00:00 AM
								5	9:00:00 AM
								8	9:00:00 AM
								12	11:00:00 AM
Temperate	NE Tas	St. Helens Is.	*Ecklonia radiata*	-41.3439	148.3428	13/12/2023	17	2	9:00:00 AM
								5	9:00:00 AM
								8	9:00:00 AM
								12	9:00:00 AM
Temperate	SE Tas	Fortescue Bay	*Ecklonia radiata*	-43.1366	147.9696	22/11/2023	14	2	9:00:00 AM
								5	9:00:00 AM
								8	9:00:00 AM
								12	9:00:00 AM

Online Resource 2

Table of nominally herbivorous fish and urchins that were included in GLMMs examing the variation in the abundance and biomass of nominally herbivorous fish and urchins. Note the term 'nominally' implies the extent to which different species remove and consume algal material varies, including across different life stages.

Realm	Species	Family	Herbivore Group	Functional Group	Reference
Tropical	*Acanthurus lineatus*	Acanthuridae	Fish	Cropper	DOI
Tropical	*Acanthurus nigrofuscus*	Acanthuridae	Fish	Cropper	DOI
Tropical	*Cetoscarus ocellatus*	Scaridae	Fish	Excavator	DOI
Tropical	*Chlorurus bleekeri*	Labridae	Fish	Excavator	DOI
Tropical	*Chlorurus microrhinos*	Labridae	Fish	Excavator	DOI
Tropical	*Chlorurus spilurus*	Labridae	Fish	Excavator	DOI
Tropical	*Diadema setosum*	Diadematidae	Urchin	Urchin	DOI
Tropical	*Echinometra mathaei*	Echinometridae	Urchin	Urchin	DOI
Tropical	*Echinothrix calamaris*	Diadematidae	Urchin	Urchin	DOI
Tropical	*Hipposcarus longiceps*	Labridae	Fish	Scraper	DOI
Tropical	*Kyphosus bigibbus*	Kyphosidae	Fish	Browser	Observed assay consumer
Tropical	*Kyphosus cinerascens*	Kyphosidae	Fish	Browser	DOI
Tropical	*Kyphosus cornelii*	Kyphosidae	Fish	Browser	DOI
Tropical	*Naso annulatus*	Acanthuridae	Fish	Mixed feeder	DOI
Tropical	*Naso brachycentron*	Acanthuridae	Fish	Browser	DOI
Tropical	*Naso brevirostris*	Acanthuridae	Fish	Mixed feeder	DOI
Tropical	*Naso lituratus*	Acanthuridae	Fish	Browser	DOI
Tropical	*Naso tonganus*	Acanthuridae	Fish	Browser	DOI
Tropical	*Naso tuberosus*	Acanthuridae	Fish	Browser	Observed assay consumer
Tropical	*Naso unicornis*	Acanthuridae	Fish	Browser	DOI
Tropical	*Naso vlamingii*	Acanthuridae	Fish	Mixed feeder	DOI
Tropical	*Parma oligolepis*	Pomacentridae	Fish	Algal farmer	DOI
Tropical	*Scarus altipinnis*	Labridae	Fish	Scraper	DOI
Tropical	*Scarus chameleon*	Labridae	Fish	Scraper	DOI
Tropical	*Scarus flavipectoralis*	Labridae	Fish	Scraper	DOI
Tropical	*Scarus frenatus*	Labridae	Fish	Scraper	DOI
Tropical	*Scarus ghobban*	Labridae	Fish	Scraper	DOI
Tropical	*Scarus globiceps*	Labridae	Fish	Scraper	DOI
Tropical	*Scarus niger*	Labridae	Fish	Scraper	DOI
Tropical	*Scarus oviceps*	Labridae	Fish	Scraper	DOI
Tropical	*Scarus psittacus*	Labridae	Fish	Scraper	DOI
Tropical	*Scarus rivulatus*	Labridae	Fish	Scraper	DOI
Tropical	*Scarus rubroviolaceus*	Labridae	Fish	Scraper	DOI
Tropical	*Scarus schlegeli*	Labridae	Fish	Scraper	DOI
Tropical	*Scarus spinus*	Labridae	Fish	Scraper	DOI

Tropical	*Scarus xanthopleura*	Labridae	Fish	Scraper	DOI
Tropical	*Siganus argenteus*	Siganidae	Fish	Cropper	DOI
Tropical	*Siganus corallinus*	Siganidae	Fish	Cropper	DOI
Tropical	*Siganus doliatus*	Siganidae	Fish	Cropper	Observed assay consumer
Tropical	*Siganus guttatus*	Siganidae	Fish	Browser	DOI
Tropical	*Siganus javus*	Siganidae	Fish	Browser	DOI
Tropical	*Siganus puellus*	Siganidae	Fish	Browser	DOI
Tropical	*Siganus punctatissimus*	Siganidae	Fish	Mixed Feeder	DOI
Tropical	*Siganus punctatus*	Siganidae	Fish	Mixed Feeder	DOI
Tropical	*Siganus spinus*	Siganidae	Fish	Cropper	DOI
Tropical	*Siganus vulpinus*	Siganidae	Fish	Mixed Feeder	DOI
Tropical	*Stegastes apicalis*	Pomacentridae	Fish	Algal farmer	DOI
Tropical	*Stegastes fasciolatus*	Pomacentridae	Fish	Algal farmer	DOI
Tropical	*Stegastes nigricans*	Pomacentridae	Fish	Algal farmer	Observed assay consumer
Tropical	*Stegastes punctatus*	Pomacentridae	Fish	Algal farmer	DOI
Tropical	*Zebrasoma scopas*	Acanthuridae	Fish	Concealed Cropper	DOI
Tropical	*Zebrasoma velifer*	Acanthuridae	Fish	Concealed Cropper	DOI
Temperate	*Aplodactylus arctidens*	Aplodactylidae	Fish	Browser	DOI
Temperate	*Aplodactylus lophodon*	Aplodactylidae	Fish	Browser	DOI
Temperate	*Centrostephanus rodgersii*	Diadematidae	Urchin	Urchin	Observed assay consumer
Temperate	*Girella tricuspidata*	Kyphosidae	Fish	Browser	DOI
Temperate	*Girella zebra*	Kyphosidae	Fish	Browser	DOI
Temperate	*Goniocidaris impressa*	Cidaridae	Urchin	Urchin	DOI
Temperate	*Heliocidaris erythrogramma*	Echinometridae	Urchin	Urchin	Observed assay consumer
Temperate	*Holopneustes inflatus*	Temnopleuridae	Urchin	Urchin	DOI
Temperate	*Olisthops cyanomelas*	Labridae	Fish	Browser	DOI
Temperate	*Parma microlepis*	Pomacentridae	Fish	Algal farmer	DOI
Temperate	*Parma unifasciata*	Pomacentridae	Fish	Algal farmer	DOI
Temperate	*Phyllacanthus parvispinus*	Cidaridae	Urchin	Urchin	DOI
Temperate	*Prionurus microlepidotus*	Acanthuridae	Fish	Cropper	DOI
Temperate	*Pseudoboletia indiana*	Toxopneustidae	Urchin	Urchin	Encyclopedia of Life
Temperate	*Temnopleurus alexandri*	Temnopleuridae	Urchin	Urchin	Encyclopedia of Life
Temperate	*Tripneustes australiae*	Toxopneustidae	Urchin	Urchin	DOI

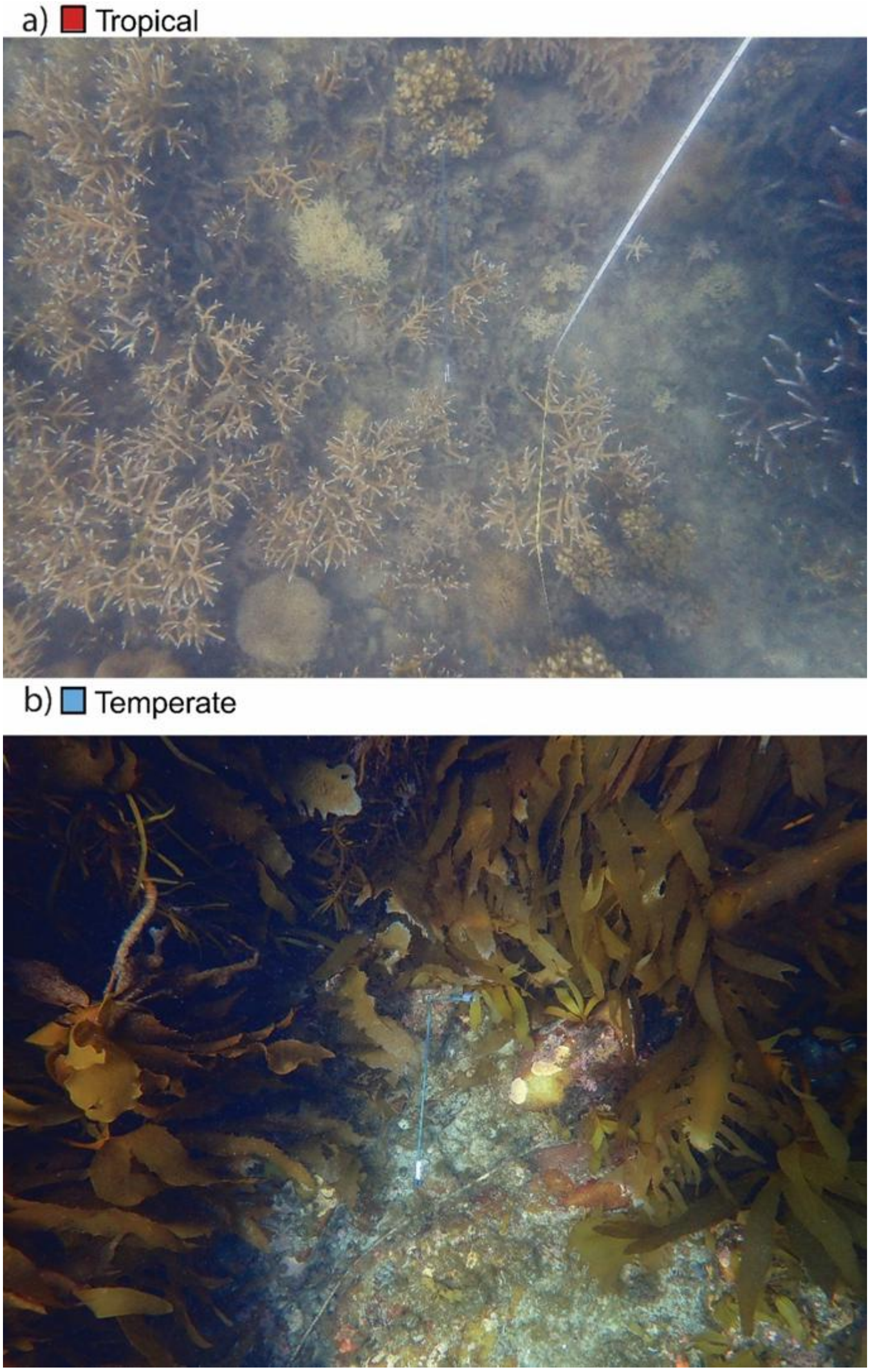

Online Resource 3 Example overhead planar photographs from which percent cover of macroalgae was determined for a) tropical and b) temperate assay deployments, with assays centred in the the camera frame, there were n = 10 photographs per depth, 100 equidistant points were overlaid on each photograph and the intercepting points of macroalgae were tallied to determine percent cover of macroalgae at each depth.

Online Resource 4 Comparison of models used to examine assay consumption (a-b) and nominal herbivore abundance and biomass (c-f). Models are compared using the corrected Akaike Information Criterion (AICc). Shown are number of parameters (k), model maximum log-likelihood (logLik), AICc, change in AICc (Δ) and AICc weight (wAICc).

Response variable		Variables	k	logLik	AICc	Δ	wAICc
a)	***Ulva* consumption (binary)**	Realm×Depth + Realm×Partitioning	12	-227.57	479.81	0	0.56
		Realm×Depth + Realm×Partitioning + Depth×Partitioning	15	-224.77	480.58	0.77	0.38
		Realm×Depth×Partitioning	18	-223.38	484.26	4.45	0.06
		Realm + Depth + Partitioning	8	-246.05	508.41	28.61	0
		Null	3	-256.91	519.87	40.06	0
b)	***Ulva* consumption (percent)**	Realm×Depth + Realm×Partitioning + Depth×Partitioning	16	-562.18	1157.54	0	0.58
		Realm×Depth + Realm×Partitioning	13	-565.89	1158.58	1.04	0.34
		Realm×Depth×Partitioning	19	-560.9	1161.47	3.93	0.08
		Realm + Depth + Partitioning	9	-585.07	1188.53	30.99	0
		Null	4	-595.94	1199.96	42.42	0
c)	**Fish abundance (across depth)**	Realm+Depth	8	27.66	-29.72	0	0.99
		Realm×Depth	11	31.87	-19.75	9.97	0.01
d)	**Fish biomass (across depth)**	Realm+Depth	8	62.95	-100.29	0	1
		Realm ×Depth	11	65.70	-87.40	12.89	0
e)	**Urchin abundance (across depth)**	Realm+Depth	8	-0.89	27.39	0	1
		Realm × Depth	11	-2.20	48.40	21.01	0
f)	**Urchin biomass (across depth)**	Realm+Depth	8	17.43	-9.26	0	1
		Realm ×Depth	11	20.55	2.91	12.17	0

Online Resource 5 Summary Analysis of Deviance results from models used to analyse assay consumption (a-c) and nominal herbivore abundance and biomass (d-k) based on type two sum of squares. Df = degrees of freedom. Models test the effects of realm, depth, and partitioning on the probability of *Ulva lactuca* being consumed for a) all sites, b) depth-gradient sites using the binary consumption response, and c) depth-gradient sites using percent-consumed, as well as the effects of realm and depth on d) fish abundance at all sites, e) fish biomass at all sites, f) fish abundance across depths and, g) fish biomass across depths, h) urchin abundance at all sites, and i) urchin biomass at all sites, only considering the nominal herbivore species outlined in Online Resource 2.

Response variable		Model used	Predictor variable	Chisq	df	*p* value
a)	***Ulva* consumption (all sites)**	Binomial GLMM (logit link)	Realm	2.837	1	0.092
			Partitioning	11.396	1	**<0.001**
			Realm × Partitioning	11.878	1	**<0.001**
b)	***Ulva* consumption (binary)**	Binomial GLMM (logit link)	Realm	1.193	1	0.275
			Depth	7.423	3	0.060
			Partitioning	3.996	1	**0.046**
			Realm × Depth	16.177	3	**0.001**
			Realm × Partitioning	16.186	1	**<0.001**
c)	***Ulva* consumption (percent)**	Beta-binomial GLMM (logit link)	Realm	1.160	1	0.281
			Depth	8.467	3	**0.037**
			Partitioning	3.982	1	**0.046**
			Realm × Depth	16.166	3	**0.001**
			Realm × Partitioning	17.258	1	**<0.001**
d)	**Fish abundance (all sites)**	Tweedie GLMM (log link)	Realm	6.847	1	**0.009**
e)	**Fish biomass (all sites)**	Tweedie GLMM (log link)	Realm	2.975	1	0.085
f)	**Fish abundance (across depth)**	Tweedie GLMM (log link)	Realm	2.298	1	0.130
			Depth	5.138	3	0.162
g)	**Fish biomass (across depth)**	Tweedie GLMM (log link)	Realm	2.927	1	0.087
			Depth	4.164	3	0.244
h)	**Urchin abundance (all sites)**	Tweedie GLMM (log link)	Realm	31.124	1	**<0.001**
i)	**Urchin biomass (all sites)**	Tweedie GLMM (log link)	Realm	50.563	1	**<0.001**

j)	**Urchin abundance (across depth)**	Tweedie GLMM (log link)	Realm<br>Depth	16.944<br>1.065	1<br>3	**<0.001**<br>0.786
k)	**Urchin biomass (across depth)**	Tweedie GLMM (log link)	Realm<br>Depth	12.789<br>1.539	1<br>3	**<0.001**<br>0.673

Online Resource 6 Summary of results from statistical models used to test the effects of realm, depth, and partitioning on the probability of *Ulva lactuca* being consumed for a) all sites, b) depth-gradient sites using the binary consumption response, and c) depth-gradient sites using percent-consumed, as well as the effects of realm and depth on d) fish abundance, e) fish biomass, f) urchin abundance, and g) urchin biomass, only considering the nominal herbivore species outlined in Online Resource 2. Model outputs are on the link-scale. SE = standard error, GLMM = generalised linear mixed-effects model.

	Response variable	Model used	Predictor variable	Estimate	SE	*z* value	*p* value
a)	***Ulva* consumption (all sites)**	Binomial GLMM (logit link	Intercept	-0.489	0.427	-1.146	0.252
			Temperate	-0.558	0.614	-0.908	0.364
			Water-column	-0.191	0.196	-0.975	0.330
			Temperate × Water-column	-1.245	0.361	-3.446	**0.001**
b)	***Ulva* consumption (binary)**	Binomial GLMM (logit link)	Intercept	-0.562	0.574	-0.978	0.328
			Temperate	0.769	0.841	0.914	0.361
			2 m	-0.228	0.391	-0.584	0.559
			8 m	-0.391	0.397	-0.985	0.325
			12 m	0.286	0.379	0.755	0.450
			Water-column	0.000	0.276	0.000	1.000
			Temperate × 5 m	-1.915	0.732	-2.614	**0.009**
			Temperate × 8 m	-0.814	0.659	-1.236	0.217
			Temperate × 12 m	-3.030	0.815	-3.716	**<0.001**
			Temperate × Water-column	-2.497	0.621	-4.023	**<0.001**
c)	***Ulva* consumption (percent)**	Beta-binomial GLMM (logit link)	Intercept	-0.904	0.550	-1.643	0.100
			Temperate	0.711	0.796	0.893	0.372
			2 m	-0.235	0.378	-0.621	0.535
			8 m	-0.399	0.385	-1.036	0.300
			12 m	0.170	0.361	0.472	0.637
			Water-column	0.017	0.265	0.062	0.950
			Temperate × 5 m	-1.821	0.697	-2.612	**0.009**
			Temperate × 8 m	-0.748	0.623	-1.200	0.230
			Temperate × 12 m	-2.882	0.783	-3.681	**<0.001**
			Temperate × Water-column	-2.474	0.595	-4.154	**<0.001**
d)	**Fish abundance (all sites)**	Tweedie GLMM (log link)	Intercept	-2.142	0.606	-3.535	**<0.001**
			Temperate	-2.345	0.896	-2.617	**0.008**

e)	**Fish biomass (all sites)**	Tweedie GLMM (log link)	Intercept	-3.940	0.614	-6.416	**<0.001**
			Temperate	-1.548	0.898	-1.725	0.085
f)	**Fish abundance (across depth)**	Tweedie GLMM (log link)	Intercept	-1.614	0.793	-2.034	**0.042**
			Temperate	-1.664	1.098	-1.516	0.130
			Depth5	-0.766	0.607	-1.264	0.206
			Depth8	-1.222	0.637	-1.917	0.055
			Depth12	-1.242	0.656	-1.893	0.058
g)	**Fish biomass (across depth)**	Tweedie GLMM (log link)	Intercept	-3.521	0.672	-5.237	**<0.001**
			Temperate	-1.522	0.890	-1.711	0.087
			Depth5	-0.253	0.630	-0.402	0.688
			Depth8	-0.470	0.639	-0.735	0.462
			Depth12	-1.385	0.707	-1.96	0.050
h)	**Urchin abundance (all sites)**	Tweedie GLMM (log link)	Intercept	-4.959	0.798	-6.217	**<0.001**
			Temperate	5.194	0.931	5.579	**<0.001**
i)	**Urchin biomass (all sites)**	Tweedie GLMM (log link)	Intercept	-6.231	0.601	-10.360	**<0.001**
			Temperate	4.931	0.693	7.111	**<0.001**
j)	**Urchin abundance (across depth)**	Tweedie GLMM (log link)	Intercept	-5.942	1.318	-4.508	**<0.001**
			Temperate	5.526	1.343	4.116	**<0.001**
			Depth5	-0.089	1.054	-0.084	0.933
			Depth8	-0.467	1.112	-0.42	0.675
			Depth12	-1.141	1.215	-0.94	0.347
k)	**Urchin biomass (across depth)**	Tweedie GLMM (log link)	Intercept	-7.772	1.536	-5.059	**<0.001**
			Temperate	5.510	1.541	3.576	**<0.001**
			Depth5	0.559	1.199	0.466	0.641
			Depth8	-0.384	1.271	-0.302	0.763
			Depth12	-0.959	1.345	-0.713	0.476

Online Resource 7 Results of post-hoc means comparisons for models examining assay consumption for a) all sites, b) depth-gradient sites using the binary consumption response, and c) depth-gradient sites using percent-consumed. Comparisons were based on a Tukey's adjustment. SE = standard error, GLMM = generalised linear mixed-effects model.

Response variable	Model used	Comparison	Estimate	SE	*z* ratio	*p* value
***a) Ulva* consumption (all sites)**	Binomial GLMM (logit link	Tropical (Benthic) – Temperate (Benthic)	0.558	0.614	0.908	0.800
		Tropical (Benthic) – Tropical (Water-column)	0.19	0.195	0.975	0.764
		Tropical (Benthic) – Temperate (Water-column)	1.993	0.642	3.106	0.010
		Temperate (Benthic) – Tropical (Water-column)	-0.367	0.614	-0.597	0.933
		Temperate (Benthic) – Temperate (Water-column)	1.435	0.304	4.725	**<0.001**
		Tropical (Water-column) – Temperate (Water-column)	1.802	0.642	2.807	**0.026**
***b) Ulva* consumption (binary)**	Binomial GLMM (logit link)	Tropical (Benthic) – Temperate (Benthic)	0.671	0.764	0.878	0.816
		Tropical (Benthic) – Tropical (Water-column)	0	0.276	0	1.000
		Tropical (Benthic) – Temperate (Water-column)	3.167	0.9	3.517	**0.003**
		Temperate (Benthic) – Tropical (Water-column)	-0.671	0.764	-0.878	0.816
		Temperate (Benthic) – Temperate (Water-column)	2.497	0.556	4.492	**<0.001**
		Tropical (Water-column) – Temperate (Water-column)	3.167	0.9	3.517	**0.003**
		**Tropical**				
		2 m – 5 m	0.228	0.391	0.584	0.937
		2 m – 8 m	0.391	0.397	0.985	0.758
		2 m – 12 m	-0.286	0.379	-0.755	0.875
		5 m – 8 m	0.163	0.404	0.403	0.978
		5 m – 12 m	-0.514	0.386	-1.333	0.542
		8 m – 12 m	-0.677	0.392	-1.725	0.310
		**Temperate**				
		2 m – 5 m	2.143	0.62	3.459	**0.003**
		2 m – 8 m	1.205	0.526	2.293	0.100
		2 m – 12 m	2.744	0.722	3.801	**0.001**
		5 m – 8 m	-0.938	0.631	-1.485	0.446
		5 m – 12 m	0.601	0.79	0.761	0.872
		8 m – 12 m	1.538	0.73	2.109	0.150
		2 m Tropical – 2 m Temperate	0.479	0.822	0.583	0.560
		5 m Tropical – 5 m Temperate	2.394	0.935	2.561	**0.010**
		8 m Tropical – 8 m Temperate	1.294	0.873	1.482	0.138
		12 m Tropical – 12 m Temperate	3.509	1.003	3.499	**0.001**
***c) Ulva* consumption (percent)**	Beta-binomial GLMM (logit link)	Tropical (Benthic) – Temperate (Benthic)	0.652	0.732	0.89	0.810
		Tropical (Benthic) – Tropical (Water-column)	-0.017	0.265	-0.062	1.000
		Tropical (Benthic) – Temperate (Water-column)	3.109	0.864	3.598	**0.002**
		Temperate (Benthic) – Tropical (Water-column)	-0.668	0.732	-0.913	0.798
		Temperate (Benthic) – Temperate (Water-column)	2.457	0.533	4.608	**<0.001**
		Tropical (Water-column) – Temperate (Water-column)	3.125	0.864	3.617	**0.002**
		**Tropical**				
		2 m – 5 m	0.235	0.378	0.621	0.926
		2 m – 8 m	0.399	0.385	1.036	0.729
		2 m – 12 m	-0.17	0.361	-0.472	0.965

5 m – 8 m	0.164	0.39	0.421	0.975
5 m – 12 m	-0.405	0.367	-1.104	0.687
8 m – 12 m	-0.569	0.374	-1.523	0.424
**Temperate**				
2 m – 5 m	2.056	0.586	3.509	**0.003**
2 m – 8 m	1.147	0.49	2.34	0.089
2 m – 12 m	2.712	0.695	3.904	**0.001**
5 m – 8 m	-0.909	0.613	-1.483	0.448
5 m – 12 m	0.656	0.779	0.842	0.834
8 m – 12 m	1.565	0.715	2.189	0.126
2 m Tropical – 2 m Temperate	0.526	0.787	0.668	0.504
5 m Tropical – 5 m Temperate	2.347	0.897	2.615	**0.009**
8 m Tropical – 8 m Temperate	1.273	0.838	1.52	0.128
12 m Tropical – 12 m Temperate	3.408	0.968	3.52	**<0.001**
